# Becoming Team Members: Identifying Interaction Patterns of Mutual Adaptation for Human-Robot Co-Learning

**DOI:** 10.3389/frobt.2021.692811

**Published:** 2021-07-06

**Authors:** Emma M. van Zoelen, Karel van den Bosch, Mark Neerincx

**Affiliations:** ^1^ Interactive Intelligence, Intelligent Systems Department, Delft University of Technology, Delft, Netherlands; ^2^ Human-Machine Teaming, Netherlands Organization for Applied Scientific Research (TNO), Soesterberg, Netherlands

**Keywords:** human-robot collaboration, human-robot team, co-learning, co-adaptation, interaction patterns, emergent interactions

## Abstract

Becoming a well-functioning team requires continuous collaborative learning by all team members. This is called *co-learning*, conceptualized in this paper as comprising two alternating iterative stages: partners adapting their behavior to the task and to each other (co-adaptation), and partners sustaining successful behavior through communication. This paper focuses on the first stage in human-robot teams, aiming at a method for the identification of recurring behaviors that indicate co-learning. Studying this requires a task context that allows for behavioral adaptation to emerge from the interactions between human and robot. We address the requirements for conducting research into co-adaptation by a human-robot team, and designed a simplified computer simulation of an urban search and rescue task accordingly. A human participant and a virtual robot were instructed to discover how to collaboratively free victims from the rubbles of an earthquake. The virtual robot was designed to be able to real-time learn which actions best contributed to good team performance. The interactions between human participants and robots were recorded. The observations revealed patterns of interaction used by human and robot in order to adapt their behavior to the task and to one another. Results therefore show that our task environment enables us to study co-learning, and suggest that more participant adaptation improved robot learning and thus team level learning. The identified interaction patterns can emerge in similar task contexts, forming a first description and analysis method for co-learning. Moreover, the identification of interaction patterns support awareness among team members, providing the foundation for human-robot communication about the co-adaptation (i.e., the second stage of co-learning). Future research will focus on these human-robot communication processes for co-learning.

## Introduction

When people collaborate in teams, it is of key importance that all team members get to know each other, explore how they can best work together, and eventually adapt to each other and learn to make their collaboration as fluent as possible. While humans do this naturally (Burke et al., 2006), it is not self-evident for robots that are intended to function as team partners in human-robot collaborations. It is well known that robotic team partners should be transparent, predictable, and explainable, but it is often overlooked that human team partners *become* predictable and explainable through a process of exploration and mutual learning.

We call the above mentioned process co-learning (Bosch et al., 2019). While existing work on human-robot collaboration and mutual adaptivity often focuses on short-term single interactions, we believe it is necessary to also look at repeated interactions to study co-learning as a mechanism for building fluent human-robot collaborations. We conceptualize co-learning as comprising two alternating iterative stages. In the *first* stage, partners observe each other and adapt their behavior to the other, leading to successful emergent team behaviors. Such adaptation can be done deliberately but often occurs implicitly and unconsciously. In the *second* stage, partners communicate about their adaptations and give each other feedback, thereby giving meaning to and becoming aware of the learned behavior. Especially this second stage of creating awareness of what has been learned helps to sustain the behavioral adaptations over time and across contexts.

We regard co-learning to be vital for creating successful human-robot collaborations. However, the term “co-learning” is relatively new in human-robot interaction literature, and it is not yet precisely defined what human-robot co-learning looks like in practice and how it should be studied. Emergent behavior can only be investigated through empirical studies; to investigate human-robot co-learning it is therefore necessary that *both* partners can learn in real-time while collaborating with each other. In this study, we therefore chose to empirically study co-learning with a human participant and a Reinforcement Learning (RL) virtual robot. For the investigation of emerging co-adaptive behaviors, we distinguish four main research questions:

1. How to identify and classify recurring behaviors that indicate co-learning in a human-robot team?
2. Which recurring sequences of these behaviors (*co-learning patterns*) can be identified, such that they can be used by the team partners to communicate about their adaptations?
3. How does the robot’s learning, emerging from the interactions, affect the human’s behavior and learning?
4. How does the human’s learning, emerging from the interactions, affect the robot’s behavior and Reinforcement Learning?

The literature on human-robot interaction and learning contains a large body of research on personalized robot tutors, in which a robot tutor learns to personalize its interactions to support the learning process of a human student, focused on classroom or training related contexts [a few examples are (Baxter et al., 2017; Gao, Barendregt, and Castellano 2017; Belpaeme et al., 2018; Vignolo et al., 2019)]. Formal training is important to initiate learning and to steer development in the right direction. However, it is important to realize that learning continues after training has been completed. Every new experience in the task provides an opportunity for human-robot teams to learn from their collaboration. An important aspect of co-learning in actual task contexts is developing and refining (shared) mental models about team members and about the task at hand, to increase mutual understanding of the best way to perform the task (Klein et al., 2004). Therefore, we specifically attempt to answer the above mentioned questions in a task context where learning happens during task execution.

In this paper, we present a behavioral study of how a human and a virtual robot, which uses a Reinforcement Learning algorithm to adapt and optimize its actions, adapt their behavior to collaboratively solve a task. We are interested in how the behavior of the human ánd the behavior of the robot changes as a result of this process, making our study fit within a new area of research in which both human and machine behavior are assessed [with their mutual dependencies; cf. (Rahwan et al., 2019)]. We first provide an in-depth elaboration of the concept “co-learning” within the context of human-robot collaboration, resulting in a definition of co-learning and the related concepts of co-adaptation and co-evolution. Based on this, we identify the requirements for empirical research into co-adaptation and co-learning, and present the design of an environment for studying co-learning. This environment has been built and used to conduct an empirical study into human-robot co-learning. From the analysis of the observed human-robot interactions, a list of patterns of adaptive interactions, and the switching between these patterns over time, were identified. These patterns can emerge in similar task contexts, forming a first description method for co-learning analyses. Moreover, it supports the creation of awareness, providing the foundation (concepts) for human-robot communication about the co-adaptation (i.e., the second stage of co-learning).

## Co-Learning: Background and Definition

Collaborative learning is a widely studied mechanism in human-only contexts, and it was Dillenbourg (Dillenbourg et al., 1996) who suggested that collaborative learning can also take place between humans and computers. Collaborative learning in the context of Dillenbourg’s work means that learning (the acquisition of new knowledge, skills, behavior, etc.) results from collaborative activities between team partners. If we look at human-robot interaction literature, several terms are used that describe a similar process in which two parties or systems change their behavior and/or mental states concurrently while interacting with each other. Co-adaptation (Xu et al., 2012; Chauncey et al., 2016; Nikolaidis et al., 2017a) and co-learning (Bosch et al., 2019) are two of them, but we also encounter co-evolution (Döppner et al., 2019), in which “co” stands for collaborative, also meaning “mutual.”

Co-adaptation and co-learning are often used interchangeably, making it difficult to understand what they stand for. There are several vision papers explaining the importance of both co-adaptation [e.g. (Xu et al., 2012; Ansari, Erol, and Sihn 2018)] as well as co-learning [e.g. (Bosch et al., 2019; Holstein, Aleven, and Rummel 2020; Wenskovitch and North 2020)], but a clear distinction between the two, or a definition specifically for co-learning, is missing from these papers. When looking at the experimental literature, however, it seems that there are subtle differences. Experimental studies on co-adaptation often focus on making the agent or robot adaptive to the human, using different kinds of information about the human [e.g. (Buschmeier and Kopp 2013; Ehrlich and Cheng 2018; Sordoni et al., 2015; Yamada and Yamaguchi 2002)]. Some studies have investigated how a human adapts in situations in which they collaborate with an intelligent agent or robot. These studies mostly focus on the performance of the human and their resulting subtle behavior change in short experiments [e.g. (Nikolaidis et al., 2017b; Mohammad and Nishida., 2008; Nikolaidis et al., 2017a)]. The studies that use “co-learning” tend to take a more symmetrical approach by looking at agent or robot learning as well as human learning, and pay more attention to the learning process and changing strategies of the human as well, often looking at many repetitions of a task (Ramakrishnan, Zhang, and Shah 2017; C.-S. Lee et al., 2020; C. Lee et al., 2018; Shafti et al., 2020). Studies on co-evolution, on the other hand, monitor a long-term real-world application in which behavior of the human as well as the robot subtly changes over time (Döppner, Derckx, and Schoder 2019).

Following these differences, we propose to distinguish the terms using three dimensions, namely 1) the time over which the development takes place, 2) the persistence of the resulting behavior/mental state over time and across contexts, and 3) the intention of the development. Table 1 shows the proposed definitions in detail. Within our research, we focus on co-learning as defined here.

**TABLE 1:** The concepts co-adaptation, co-learning and co-evolution defined in terms of timespan in which they occur, persistence and intention.

	Co-adaptation	Co-learning	Co-evolution
Timespan	Short (seconds*—*hours)	Medium (hours*—*weeks)	Long (weeks*—*years)
Persistence	Developed behavior/mental state does not necessarily persist over time, and probably not at all across contexts	Developed behavior/mental state persists over time and possibly across contexts	Developed behavior/mental state might persist for a while but possibly continues to evolve, similar to the development of habituation
Intention	Changes and developments happen as a consequence of interactions and an implicit or explicit drive to improve performance or experience	Explicitly goal-driven: Attempts to improve performance or experience; learning is an explicit goal	Changes and developments happen as a consequence of interactions and possibly an implicit drive to improve performance or experience

In a human-robot co-learning process, a human and a robot collaborate on a given task. In order to do well on the task, they need to learn all kinds of implicit and explicit knowledge related to both the task itself as well as the collaboration and interaction between them. Related to the task they can, for example, learn the technical details of how the task should be executed. Related to the collaboration, they can, for example, learn social collaboration skills. Related to both, they can learn about their own role and the role of the other in the task and the consequences of their own and their partner’s actions and mental state on the task (how to collaborate in context of the task). Ultimately, learning this should help them to together perform well on the task, to build understanding of each other in context of the task and to calibrate the trust that the human and the robot have in each other. We focus our work on this last type of combined task and collaboration learning.

We further define co-learning to be comprised of two stages that follow each other in continuous iterative cycles, namely 1) co-adaptation, and 2) a communication process. Part (a) is therefore a process in which team members (sometimes unconsciously) adapt to each other and the task, thereby changing and developing their behavior as a consequence of interactions and an implicit or explicit drive to improve performance or experience (see again Table 1). Part (b) is a process in which these implicitly developed behaviors are shared and discussed through direct communications or interactions between team members, thereby making the team members aware of the implicit adaptations. This combination ensures that learned strategies are grounded in the context and task and can be strategically used in new contexts.

## Research Challenges

Many research challenges follow from the conceptualization of co-learning, due to the fact that both human and robot are non-static. They are both constantly developing, changing and adapting, and they influence each other in the process. This means that it is not possible to study only one of the team partners; it is necessary to take a symmetrical approach, where both human and robot are studied through the interactions between them. Moreover, co-learning in dynamic tasks is a continuous process in which new task situations that appear dynamically require new learning over and over again. Therefore, focusing on one specific interaction, or on team performance as end result, does not offer a complete picture. We need to study all interactions that contribute to this process. These specific dynamic properties need to be taken into account in the design of experiments, as well as in the analysis and discussion of results. Following from this, and to provide a broader view on the specific study that we present in this paper, we have defined three research directions that need to be addressed in the study of human-robot co-learning:

1. **Research into enabling and assessing co-learning:** to understand the dynamics of co-learning, we need to investigate what kind of behaviors and interactions drive co-adaptation and co-learning, and how learning processes of human and robot team members influence each other.
2. **Research into interaction patterns that make team partners explicitly aware of learned behavior, such that behavior can be sustained over time and context:** in order to create sustainable team behavior, human and robot need to communicate about learned behavior to ensure that they are aware of useful learned behavior. It is important to investigate what kind of communication interaction patterns enable this specific type of communication.
3. **Research into a dynamic team mental model that takes into account naturally occurring changes in interaction patterns, and how such a model can support the robot in its learning process:** as humans, we are able to anticipate on the fact that our team members learn and change. It is important to investigate how a dynamic team model can enable robots to also anticipate the fact that their human team member is continuously changing.

The study presented in this paper focuses on the first research direction; the research questions presented in the introduction have been derived from it. More specifically, in the experiment that we describe in the following sections, we have chosen to focus on the first stage of the co-learning process: co-adaptation as a precursor for co-learning. We do not yet address questions concerning communication about learned behaviors (research direction 2), but focus on the implicit behavioral adaptations that occur within a relatively short time span. It is expected that results of the present study will provide pointers for how to investigate the issues associated with research challenges two and three above.

## Research Environment: Designing Task, Agent, Context

### Context

To study co-learning in human-robot teams, a suitable task context needs to be designed. We identify the following requirements for such a task context in which we can study co-adaptation according to the definition in Table 1:

1. It should be possible for the team to improve its performance by making effective use of the capabilities of the individual team members [as this is necessary to make it a team task (Johnson et al., 2014)];
2. There should be possibilities for implicit adaptation and learning for both human and robot team members;
3. It should accommodate different emergent collaborative strategies for solving the task;
4. For this first study, the task and team work should be simple enough for a Reinforcement Learning agent to learn new behavior in a short number of rounds, such that we can study co-adaptation in relatively short experimental sessions;
5. To ensure societal relevance of this research, the task should be based on a real-life domain in which there is a need for autonomous robots that function as team partners.

As a general context for defining a task, we chose Urban Search and Rescue (USAR). A lot of research on human-agent teaming is done in USAR-related tasks (Lematta et al., 2019), because the safety-critical nature makes the application of human-robot collaboration very useful; there are ongoing initiatives which aim to use robots in real USAR teams (requirement 5). Moreover, it is a dynamic task context with many possible subtasks and possibilities for the introduction of threats, safety risks and changing information.

**FIGURE 1:**
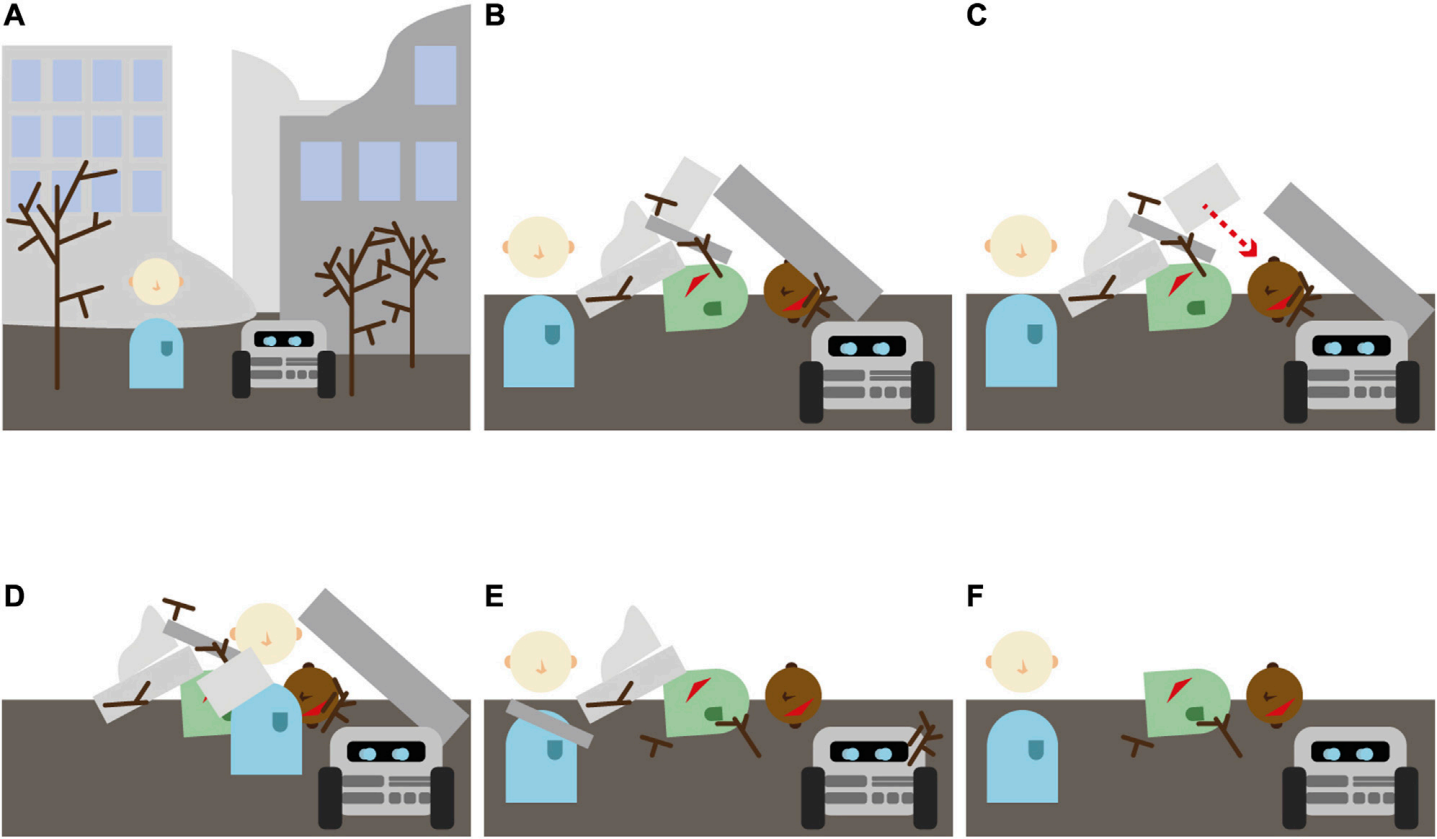
A storyboard describing how a human-robot team might free an earthquake victim from underneath a pile of rocks. In this storyboard, we can see how the robot picks up a large rock, unaware that this will cause another rock to fall on the head of the victim **(panel C).** The human notices the issue, and steps in to prevent the rock from falling **(panel D).** This event can help the robot learn about the task, and that it apparently made a mistake. The human can learn about the capabilities of the robot, namely that it didn’t understand how the rocks would fall and that it would cause harm.

We developed an earthquake scenario for our human-robot USAR team, with the team’s task to remove rubble and debris from a victim. To get a better understanding of the task, and of the knowledge and capabilities it requires from partners, we created a storyboard (Figure 1). The storyboard shows a possible scenario in which the robot picks up a large rock to clear it away, not realizing that the action may lead to a small rock falling on the head of the victim (Figure 1C). When the human notices this, this provides an opportunity to jump in, and to prevent the rock from crushing the victim (Figure 1D). This event facilitates the human learning that the robot apparently does not understand the risks of falling rocks. It provides the robot with the opportunity to learn that it made a mistake. The event furthermore provides an opportunity for team members to communicate about the event, their actions, and to plan how they will manage such situations together in the future. This storyboard illustrates that using the unique capabilities of both the human (insight into strategic choices) and robot (physical strength) can be exploited to achieve better task performance (requirement 1). The task of removing rubble from a victim allows for a great diversity in task planning and execution, and for the development of individual strategies (requirement 2 and 3), as the different debris can differ in shape, size and location, while enabling simple basic actions to create strategies for solving the task (requirement 4).

### Task Implementation

**FIGURE 2:**
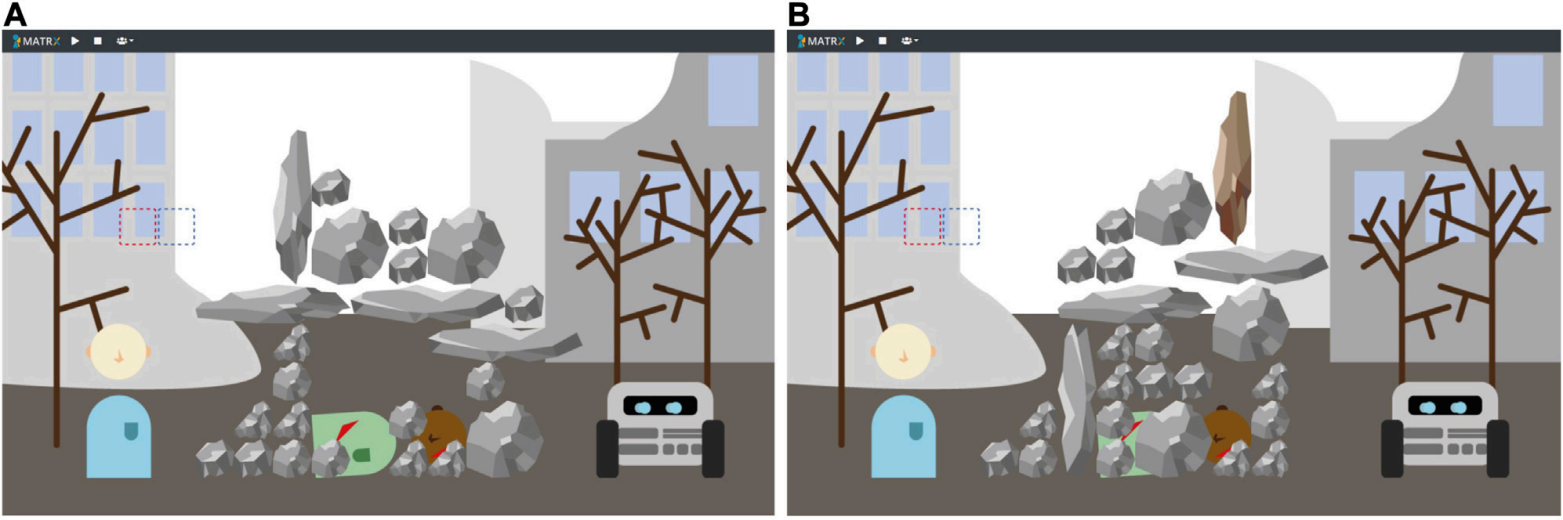
The USAR task environment programmed in MATRX. It shows a victim underneath a pile of rocks, and a human and a robot representing the team members. The dashed red square (above the human’s head) represents the hand of the human that can be moved to pick up rocks. The dashed blue square represents the hand of the robot. Scene **(A)** was used as level 1 in the experiment, while scene **(B)** was used as level 2.

We developed a digital task simulation of the described USAR context using Python and the MATRX package (MATRX Software, 2020). MATRX is a package for rapid prototyping of human-agent team environments, which supports easy generation of an environment, object and agents. Figure 2 shows a screenshot of the simulation. The scene involves three characters: a victim buried underneath a pile of rocks (shown in the middle), an explorer (avatar on the left, played by a human participant) and a Reinforcement Learning robot agent (avatar on the right). The goal of the task is to free the victim by clearing away all rocks that are in front of the victim, as well as to create a pathway to the victim from either the left or the right side. In order to score well, this must happen as quickly as possible, and no additional rocks (or as little as possible) should fall on top of the victim, as that will cause extra harm. Both the human and the virtual robot each have a set of actions they can perform, such as picking up rocks and dropping them somewhere else. However, the extent to which they can perform actions differs: the robot can pick up large and small objects, and break large objects into pieces. Humans can only pick up small objects. Humans however have a better insight in certain aspects of the task that dictate which actions are useful to do, such as how rocks will fall when other rocks are removed or replaced. This insight stems from the fact that humans have “common sense,” which helps us understand the probable consequences of actions. In order to complete the task successfully participants must collaborate with the virtual robot (requirement 1), while managing their actions in such a way that the robot does not accidentally drop rocks on the victim’s head. Since it is not clear at the start what the best strategy would be to solve the task quickly, both partners need to learn and adapt as they go (requirement 2). The levels are designed such that there are different possible ways to solve the task (requirement 3), and it is a discrete environment build on a simple state machine, making it possible to design a Reinforcement Learning agent that can process the environment (requirement 4).

### Learning Agent

To be able to empirically study how a human and a robot co-adapt while collaborating on a task, the robot should be able to try out and evaluate different actions, to be able to choose the policy that best fits the goals of the team given the adaptations done by the human team member. We chose to use Reinforcement Learning to enable the agent to learn, for three main reasons:

1. The robot had to be able to learn real-time, on the basis of rewards: as the behavior of the human team partner is adaptive and unpredictable, we cannot determine optimal behavior before the start of the task. This means that the best way to find the optimal strategy for solving the task collaboratively would be to get feedback or rewards on performance.
2. The described task can be solved by a sequence of actions that manipulate the state of the world with each action, therefore, the learning algorithm had to be able to learn a sequence of actions given different sequential task situations.
3. The described task is a human-robot scenario, but it can also conceptually be seen as a multi-agent scenario as both the robot and the human are autonomous and learning agents within the collaboration. Reinforcement Learning is an often used and widely studied mechanism in multi-agent scenarios, for reasons related to reasons one and two above as well [see e.g. (Kapetanakis and Kudenko 2002; Foerster et al., 2016)].

**FIGURE 3:**
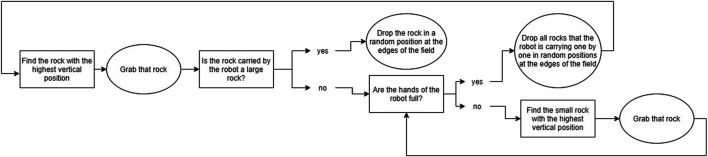
A flowchart showing the rule-based decision making the agent would go through when using Macro-Action 1.

**FIGURE 4:**
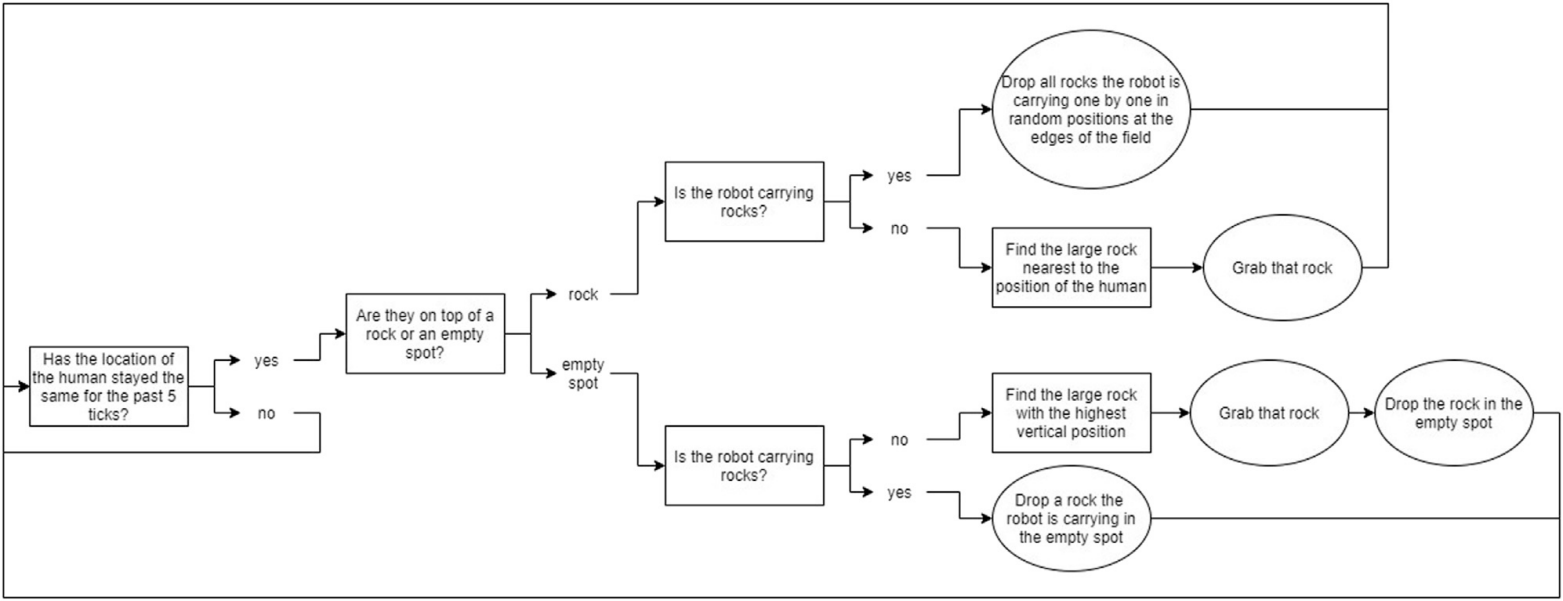
A flowchart showing the rule-based decision making the agent would go through when using Macro-Action 2.

**FIGURE 5:**
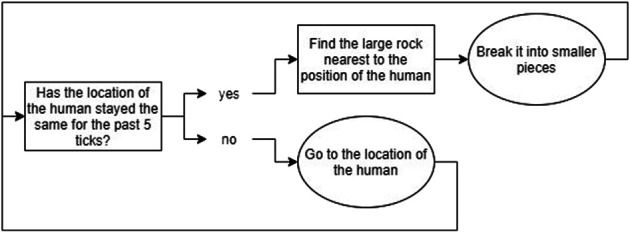
A flowchart showing the rule-based decision making the agent would go through when using Macro-Action 3.

**FIGURE 6:**
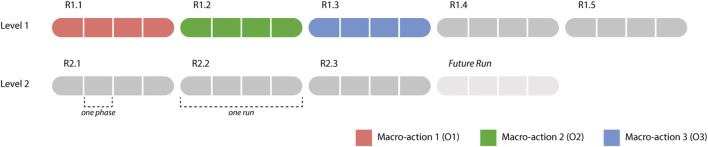
An overview of the representation of the learning problem embedded in the experiment. It shows the different runs that a participant went through (5 runs for level 1, 3 runs for level 2), as well as how the runs were separated into 4 phases defined by the Phase Variables. The colors show how in R1.1, R1.2 and R1.3, the robot usually used *O1—picking up all*, *O2—passive large rocks* and *O3—breaking* respectively in each phase. From R1.4 onwards, the robot would choose a Macro-action based on the learned Q-values. The Future Run portrays the behavior that the robot would engage in if there were another run, based on the Q-values *after* R2.3.

Reinforcement Learning has been designed for learning sequences of actions in tasks that can be modeled as Markov Decision Processes (van Otterlo and Wiering 2012), in which the transitions between states are unknown. In contexts where agents collaborate and learn with a human, these transitions are unknown since it is unknown what the human will do; this is also the case in our task. While such a human-agent collaborative context poses many challenges (e.g. large state spaces, long convergence times and random behavior in the beginning) (Dulac-Arnold, Mankowitz, and Hester 2019), earlier work has shown that RL can be used successfully for learning behavior in real time when interacting with a human, provided that the learning problem is simple enough (Weber et al., 2018). Since we used RL mostly as a tool to ensure that the agent could adapt over time, and not as a goal in itself, we created a RL mechanism that is much simpler than the current state-of-the-art, but that would provide the basic learning that is sufficient for our research goals. We simplified the task by modeling it as a semi-Markov Decision Process (Sutton, Precup, and Singh 1999), which means that the task is divided into several “phases,” which serve as the states in the RL algorithm. Normally states last one timestep, whereas in a semi-Markov Decision Process, these phases can last variable amounts of time. Our state definition describes the state of the environment based on the amount of rocks present in the area around the victim. The state space is defined by S = (Phase 1, Phase 2, Phase 3, Phase 4, Goal Phase). Table 2 describes the details of the individual states. We chose to not explicitly represent learning about collaboration in the learning agent, since we wanted to focus on implicit behavioral adaptations (as explained in *Research Challenges*). We combined this with a system inspired by the Options Framework (Stolle and Precup 2002) and a basic greedy Q-learning algorithm. In the Options Framework, agents use RL to learn a meta-policy as well as several “sub-policies.” These sub-policies can also be seen as macro-actions; they are combinations of atomic actions that are used together to solve parts of the task. Usually, these macro-actions are learned in parallel with the meta-policy, but sometimes they are pretrained, such as for example in (Illanes et al., 2019). To further simplify the learning problem, we chose to predefine three rule-based macro-actions; the agent could choose from these macro-actions in each phase of the task (a description of each macro-action is given in Figure 3, Figure 4, and Figure 5). The rewards for the RL algorithm are based on two factors: 1) the time it took the team to move to the next phase, and 2) the amount of additional harm done to the victim. The agent would receive this reward when transitioning into a new phase, or when the task terminates due to becoming unsolvable or due to a timeout. The height of the rewards was made such that the total reward given was always negative. With initial Q-values of 0, this ensured that in the first three runs of the experiment, the agent would try out all three macro-strategies in order, to enforce initial exploration. A visual overview of the learning problem is provided in Figure 6, after we have explained more details about the experimental method.

**TABLE 2:** The task conditions specified for each Phase Variable used in the state space of the Reinforcement Learning algorithm.

Phase	Description
Phase 1	The starting phase: Describes the state of the task environment when no rocks have been moved
Phase 2	The heights of all piles of rocks added up is now at least 10 rocks lower than in phase 1
Phase 3	Phase 2 has been reached, and the heights of all piles of rocks added up is now at least 20 rocks lower than in phase 1
Phase 4	Phase 2 and 3 have been reached, and either there are no more rocks directly on top of the victim, OR one of the sides of the task field is cleared from rocks, meaning there is an access route to the victim from either the left or right side
Goal phase	Phase 2, 3 and 4 have been reached, and there are no more rocks directly on top of the victim, AND one of the sides of the task field is cleared from rocks, meaning there is a free route from either the left or right side to the victim. The task terminates when this phase is reached

### Claims: Expected Observations

We expect to observe several behaviors within this task environment, given that it was designed to study co-learning behavior. We have formulated these expected observations as the following claims:

• Different participants develop different ways of performing the task;
• The agent learns different sequences of macro-actions for different participants;
• Different teams converge to different ways of performing the task;
• The agent converges to a specific sequence of macro-actions for most participants;
• The human converges to a specific strategy within the experiment.

In the Discussion (*Discussion*), we use the results of our experiment to critically evaluate whether we have been able to study co-adaptation as a precursor for co-learning with our methods by verifying to what extent these claims hold.

## Method of Study for Identifying Interaction Patterns

The experimental setup and procedure described below was approved by the Human Research Ethics Committee at Delft University of Technology on August 17th^,^ 2020 (reference number: 1261).

### Participants

A total of 24 people participated in the experiment (17 female, seven male), recruited through personal connections on LinkedIn, within the university, from a Slack community on AI and Design and from interns at TNO. The average age among the participants was 24.8 (Std = 2.47). All of the participants had a university degree in a STEM field. Most of them had little to some experience with gaming (n = 7 for “little experience,” n = 10 for “some experience”). Also, most of them expressed that they had no to little experience with human-robot collaboration (n = 11 for “no experience,” n = 7 for “little experience”) or human-robot collaboration research (n = 11 for “no experience,” n = 5 for “little experience”).

Due to a few problems in the data collection, some participants were excluded from all of the analyses or some of the analyses. Two participants (one female, one male) were excluded from all analyses, because there were significant connectivity issues during the execution of the experiment and/or data collection went wrong on more than one factor. One participant (female) was excluded from the questionnaire analyses, because their data was not properly saved, and one participant (male) was excluded from the robot behavioral analyses, because the log data was not properly saved.

### Design and Materials

Participants were divided over two conditions: 1) a condition in which participants were instructed to think aloud and 2) a condition in which they were asked to perform the task in silence. Since we study learning processes, and since it is known that thinking aloud can have an effect on learning, the two conditions ensured that we had control over any possible effect.

We presented the task environment described in *Task Implementation* to all participants, in the form of two different levels. The first level was designed to be relatively easy, as it could be solved by simply clearing away all rocks (Figure 2A). A complicating factor was that breaking rocks would easily hurt the victim, which would therefore need to be avoided. The second level was designed to be more challenging: it contained a brown rock that could not be picked up at all (Figure 2B). This means that if the brown rock would fall on top of the victim, it would no longer be possible to save the victim and finish the task.

Participants played the first level five times, as it was estimated from pilot runs that five times would provide ample opportunity for both the participant and the robot to learn a working strategy. Participants then played the second level three times, to give the team the opportunity to adapt to the new situation. The repetition allowed for within-subject analyses, in which the behavior of participants could be compared between rounds, as well as between-subjects analyses of learning. For an overview of how the definition of the task in the Reinforcement Learning algorithm combines with this setup, Figure 6.

### Procedure

The experiment was conducted through a video call between the experimenter and each individual participant, while both were located in their own home for the course of the experiment. Participants were given access to the experimental task using Parsec, which is a screen-sharing platform made for collaborative gaming (Parsec., n.d.). This ensured that participants had control over the task environment, while allowing the experimenter to observe their behavior.

All participants went through the following steps:

1. Participants were seated in front of their own computer at home;
2. They read the instruction, signed the consent form and provided some demographic information as well as information on their experience with video games, human-robot collaboration and human-robot collaboration research;
3. Participants had the opportunity to do a short test scenario of the task without the virtual robot, to familiarize them with the task environment and the controls;
4. Participants were presented with the first pre-specified level. After five runs, the new level was presented to the participant, which they played three times;
  a. The participants in condition A were asked to think aloud during the execution of the levels;
  b. After each level, participants completed a selection of the questionnaire on Subjective Collaboration Fluency [taken from (Hoffman 2019), see Supplementary Appendix SA for the questions used]. In addition, they were asked to rate how confident they were that their strategy was a good strategy for solving the task on a scale of 1–10;
  c. After the first five runs and at the end of the experiment the participants were interviewed about their experiences.

### Data Collection and Analysis

Several types of data were collected in order to answer our research questions:

1. Screen captures and notes of behavior in the MATRX environment during the execution of the experiment
2. Voice recordings of the participants in condition A while they are thinking aloud during the execution of the experiment
3. Voice recordings of short interviews (see Supplementary Appendix SA for the questions asked)
4. Collaboration Fluency scores
5. Confidence of Strategy scores
6. Q-table as learned by the robot and log of how it changes

We will explain in more detail how this data was collected and how it relates to our research questions in the sections below.

#### Behavior

In order to identify what interaction patterns drive co-adaptation and co-evolution, we wanted to look at how the behavior and strategy of the team changed over time, and which interactions were used in that process. We used data types 1, 2, 3 and 6 for this. The screen captures and notes (data type 1) serve mainly as data on the human behavior, while the thinking aloud output and the interviews (data types 2 and 3) help to explain why humans behave in a certain way. The Q-tables (data type 6) serve to see what strategy the robot chose in each phase of the task. Normally, behavior of a robot driven by RL is assessed by looking at the cumulative rewards. As we are not necessarily interested in performance, but in the behavior resulting from the learning process [as prescribed in (Rahwan et al., 2019)], we chose to look at the development of the Q-tables, to understand what macro-action the robot learned to choose in each phase.

A Grounded Theory (Charmaz 2014) process was used to identify recurring adaptive behaviors from the screen captures and notes. This means that we went through a process of open coding first, while constantly writing short memos of observed patterns. After that, we collected all codes and categorized and clustered them until reaching the desired level of detail.

We will explain how the behavioral data and Q-tables were used to answer our research questions in more detail in *Results*.

#### Subjective Collaboration Fluency and Confidence Score

Within the task that we designed, it is quite difficult to keep track of task performance due to the possibility for large differences in strategies, as well as because the task can become unsolvable. To still keep track of how the human-robot team performed over the course of the experiment, we have chosen two measures for tracking subjective task performance: subjective collaboration fluency and confidence score (data types 4 and 5). These measures helped us to validate that our experiment setup actually allowed for learning and improvement.

For subjective collaboration fluency, we used a short version of an existing questionnaire (Hoffman 2019). To measure participants’ confidence in their strategy, we asked them to rate confidence on a scale from 1 to 10 with the following question: “How confident are you that your strategy is the right strategy?”

## Results

### Subjective Collaboration Fluency and Confidence Score

**FIGURE 7:**
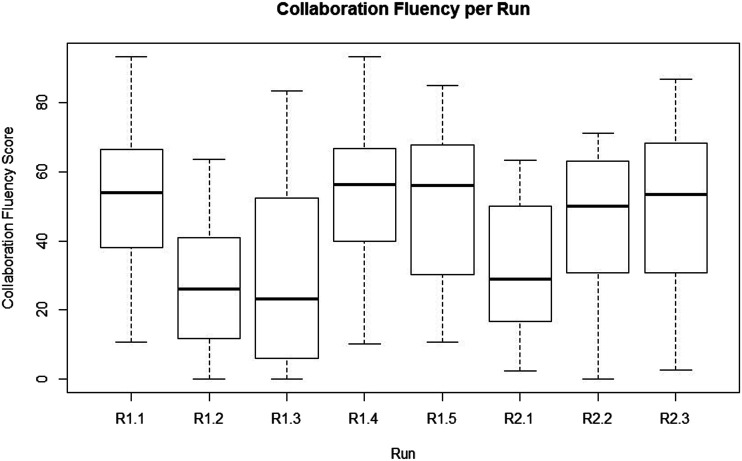
The Collaboration Fluency scores per run in the experiment for all participants.

We have created a box plot of the Subjective Collaboration Fluency scores (Figure 7). The Confidence scores followed a very similar pattern, therefore we do not go into further detail about those. Both scores follow a pattern with scores starting off relatively high in run one, after which they drop for run two and three, move up again for four and five, drop again for six and then move up for the last two runs. To test whether thinking aloud and the number of the run affected participants’ experience of collaboration with the robot, the Subjective Collaboration Fluency score was entered in a one-way repeated measures ANOVA with Thinking Aloud (yes/no) as between-subjects factor, and run number (1–8) as a within-subjects repeated measure factor. Results show that there was no significant difference between the participants who were instructed to think aloud (Mean = 40.56, Std = 22.78) and those who were not (Mean = 45.12, Std = 25.75) (F = 0.81, *p* = 0.38), while there was a significant effect on the run (F = 5.97, *p* < 0.0001). When looking at Figure 7, we expected this significant difference to exist between round one and round two, round three and round four, round five and round six and round six and round seven (scores went down after round one, up after round three, down again after round five and up after round six). To test whether these differences between rounds were significant, we did a post-hoc analysis using a Tukey HSD test, which mostly confirmed the differences visible from the plot: R1.1 and R1.2 are significantly different (*p* = 0.006), R1.3 and R1.4 are significantly different (*p* = 0.006), R1.5 and R2.1 are almost significantly different (*p* = 0.058) but R1.4 and R2.1 are (*p* = 0.003). R2.1 and R2.2 did not differ statistically, but R2.1 and R2.3 do, although not significantly (*p* = 0.114).

The pattern of scores on both fluency and confidence over runs are probably caused by the setup of our experiment. In the first run, the robot would use macro-action 1 for the whole task, which is the easiest to work with from a participant perspective. Therefore, participants may have been inclined to assign high scores in the beginning. In run 2 and 3, the way the RL algorithm is implemented causes the robot to use macro-action 2 and 3 respectively for the whole task, which are quite hard to understand from a participant perspective, arguably leading to a lower level of experienced fluency. In run 4 and 5, the robot would start picking its macro-action based on previous performance, while the participant would have learned to work with the robot a bit more. It is likely that this made the fluency scores go up again. Run 6, however, introduced the new scenario in which previously learned strategies often did not work anymore. In runs seven and eight the human-robot team would then learn to perform better at this second scenario, inducing participants to give higher scores on experienced collaboration fluency. Following this explanation of the scores, these results suggest that our experiment design indeed allowed for a learning process of the human-robot team as we anticipated.

### Interaction Pattern Analysis

The open coding process of behavioral summaries, based upon the information from videos, interviews and notes, yielded a list of 52 different behaviors. These behaviors consist of *task-related actions* by the participant; *interactions* between the participant and the virtual robot; *learning* (participant learns something about the task or the collaboration); *strategies* (combinations of actions executed over longer periods of time); *team performance* and participant *emotional responses*. After excluding behaviors that did not relate to adaptation in specific (e.g. actions such as “picking up top rocks”) and behaviors that were more of an assessment of the quality of a behavior rather than a description (e.g. performance factors such as “not understanding the link between waiting and robot action”), a list of 38 behaviors was left.

These 38 behaviors were categorized in the following two categories [based on the categorizations made in (van Zoelen et al., provisionally accepted)]:

• Stable situations (9 behaviors): behaviors observed *in-between* adaptations, such as the behavior of the participant alternating acting and waiting for the robot.
• Sudden adaptations (29 behaviors): behaviors in which the human and/or robot adapted their actions, thus starting a transition from one stable situation to another. The adaptation happens in a single moment or over a short period of time, often in response to a newly hypothesized or discovered property of the partner’s behavior.

The full list and categorization can be found in Supplementary Appendix SB. The behaviors listed in the Appendix are closely tied to the experimental task. The descriptions of the behaviors were processed to fit co-adaptation in general (such that we can call them interaction patterns). For this purpose, some of the behaviors were combined into one descriptive interaction pattern. The resulting list, consisting of 23 interaction patterns (five stable situations, 18 sudden adaptations), is presented in Table 3.

**TABLE 3:** The interaction patterns identified from the behavioral data, including a description of what they entail.

Category	Concept	Description
Stable situation	Actively synchronizing actions with a team member	Human understands the capabilities of another team member and actively uses their own actions to make optimal use of the combined capabilities
	Alternating actively working on the task and waiting for a team member	Human switches between performing their own task for a while, then waiting for a team member to perform their task, and so on
	Being generally passive and letting a team member do most of the work	Human is overall passive and lets the other team member do the work
	Damage control: Prevent damage caused by a team member	Human performs actions that prevent their team member from causing intentional or unintentional harm or damage
	Focusing on own task	Human performs their own task without paying much attention to their team member
Sudden adaptation	Avoiding communication with a team member	One of the team member actively avoids the other team member to avoid unwanted communication interpretations
	Being confused by non-human-like behavior	A human team member is confused by non-human-like behavior performed by a team member
	Being confused by unexpected behavior (negative)	One of the team members is confused or frustrated by behavior performed by their team member that they did not expect
	Being happy that a team member does as expected	One of the team members is happy that their team member performs the kind of behavior that they expect and hoped for
	Being surprised by unexpected behavior (positive)	One of the team members is positively surprised by behavior performed by their team member that they did not expect
	Coming into action when a team member comes into action	A team member starts to actively perform their task after a period of inaction, when their team member also starts to actively perform their task after a period of inaction
	Doing useless or harmful actions because there is nothing else to do	A team member is unable to perform useful actions, therefore starts performing useless or harmful actions
	Feeling alone, as if team member does not help	A human team member feels left alone
	Following a team member’s action	A team member follows or copies the action performed by another team member
	Learning about behavioral cues	A team member gains insight into specific behavior performed by another team member
	Learning about own capabilities	A team member gains insight into their own capabilities
	Learning about team member’s capabilities or strategy	A team member gains insight into the capabilities or strategy of another team member
	Moving around different task components	A team member moves around different task components without actually performing any task
	Team member changes strategy, which is visible by a behavioral cue	A team member observes that another team member changes strategy by a behavioral cue
	Team member performs an action that makes no sense	A team member performs a useless action
	Trying to communicate by interacting with a team partner	A team member attempts to communicate with another team member by directly interacting with them, for example by coming close to them
	Trying to communicate by signaling task actions	A team member attempts to communicate with another team member by trying out different actions that they want their team member to perform
	Waiting for a team member to start acting	A team member waits for another team member to start performing their task

The biggest group is that of “sudden adaptations,” the patterns that often arise in response to a discovery, an expectation, or a surprise of one of the partners. In order to better understand this important group of adaptive interaction patterns, we explored in more detail the nature of the triggers that initiate them, what characterizes the execution of these patterns, and what they bring about in the human-robot collaboration. Again following the approach taken in [van Zoelen et al. (provisionally accepted)], we used the following terms to describe the sudden adaptations:

• External trigger: an event outside of the partner (e.g. in the task, environment or other partner) triggers an adaptation to a new stable situation;
• Internal trigger: an event inside of the partner (e.g. a specific expectation or change of mind) triggers an adaptation to a new stable situation;
• Outcome: a specific action that is preceded by an internal or external trigger, that will gradually develop into a new stable situation afterward;
• In-between-situation: a specific action that is preceded by an internal or external trigger, that serves as a new trigger for adapting to a new stable situation afterward.

The results of this can be found in Supplementary Appendix SB.

### Collaborative Learning

In addition to developing a comprehensive description of adaptive interaction patterns, we further explored how human behavior, and specifically human behavior adaptation, influenced learning by the virtual robot. We analyzed and coded human adaptive behavior at a detailed level by identifying the interaction patterns as described above, but in order to analyze how the development of robot behavior and human learning depend on each other, a different level of detail was necessary. We looked at three aspects of the data:

• For each participant, we looked at the Q-tables of the virtual robot at the end of each run in the experiment, to see which of the three macro-actions received the highest expected reward in the different phases and runs of the task;
• For each participant, we identified the main behavioral strategy used by the human per run, as well as during the whole experiment;
• We analyzed how the chosen macro-actions of the virtual robot can be associated with specific behavioral strategies of the participants.

We will describe our process for all three of these aspects in more detail below.

#### Virtual Robot Q-Tables

**FIGURE 8:**
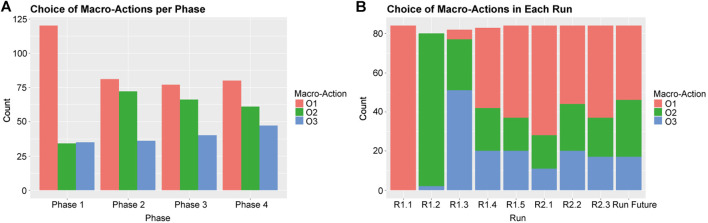
An overview of how often certain Macro-actions were chosen by the robot across all participants per phase **(A)** and per run **(B)**.

The Reinforcement Learning algorithm addressed learning when to apply which of three macro-actions or options (as described in Figure 3, Figure 4, and Figure 5; we will call them *O1—picking up all*, *O2—passive large rocks* and *O3—breaking* from here onwards) and used four phase variables to identify states. Figure 8 shows an overview of how often the robot learned to pick specific macro-actions in each phase (Figure 8A) and each run (Figure 8B), based on the macro-action with the highest expected reward.

As the robot’s choice for macro-options is not clearly related to the phases in the task (especially phase 2, 3 and 4 are very similar, as can be seen in Figure 8A), we looked mostly at Figure 8B to understand how the robot’s behavior developed. The figure shows that in the first three runs the robot mostly tried out all macro-strategies one by one, as determined by how the algorithm was programmed. Small deviations from this are likely caused by some participants going back and forth between phases in the task, rather than moving through them linearly as we initially expected. The robot generally learned to select *O1—picking up all* most of the time for most participants over the course of the next few runs, which fits with how level 1 of the experiment was designed. From run 6 onwards, when the second level was introduced, the robot learned to choose *O2—passive large rocks* and *O3—breaking* more often. This shows that the robot is able to generally learn what works best for the task.

#### Participant Behavioral Clustering

To better understand how the behavior of the participants developed over time, we performed a manual clustering of participant behavior per run. Based on the behavior observations as described in *Interaction Pattern Analysis*, we defined the following behavioral clusters:

• Just focus on own behavior efficiently
• Balancing acting and waiting
• Exploring how the robot works by observing and trying to communicate
• Actively using *O3—breaking*
• Actively using *O2—passive large rocks*

**FIGURE 9:**
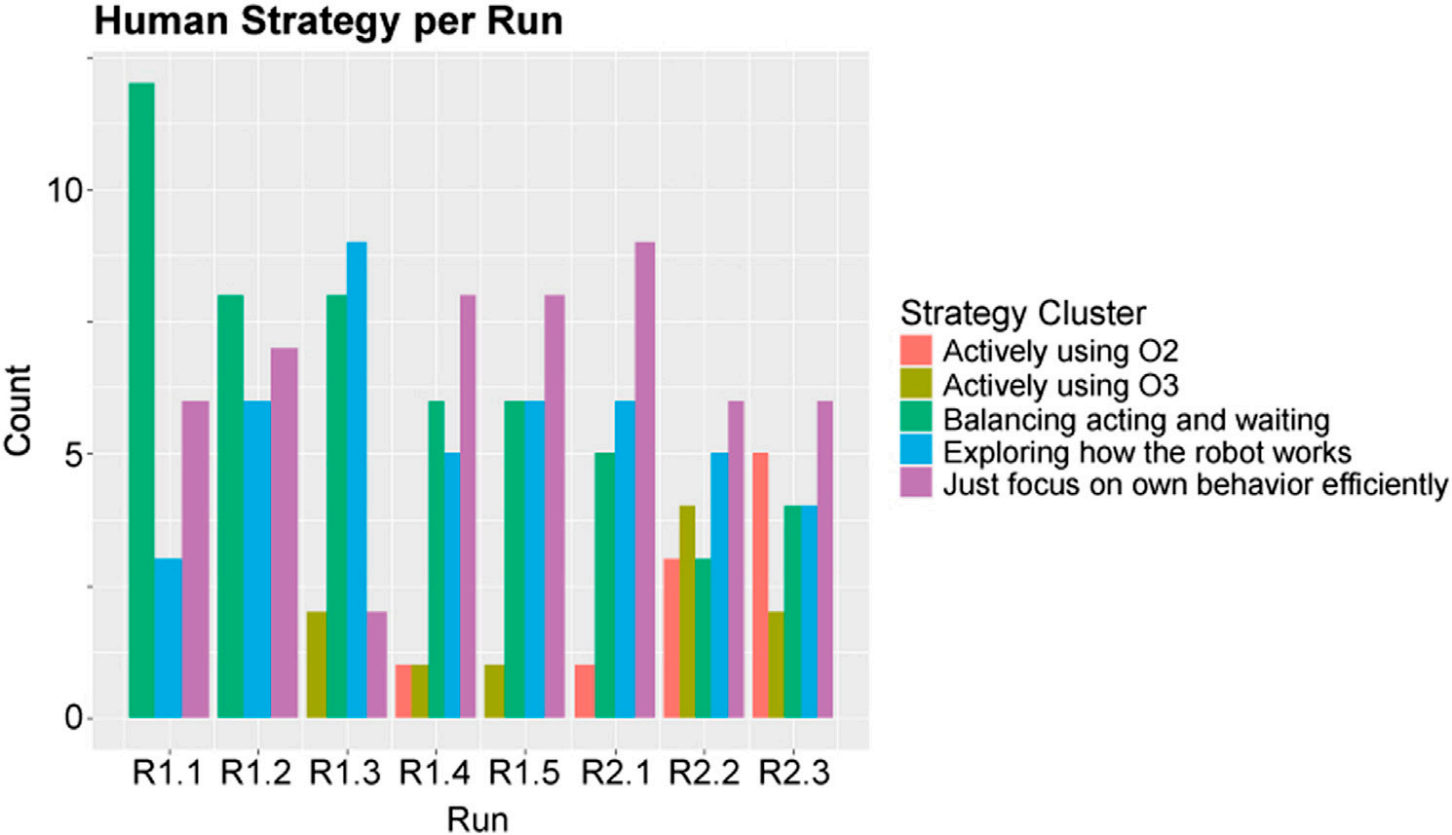
An overview of how many participants used specific behavioral strategies per run.

The result of this clustering can be seen in Figure 9. It is difficult to find detailed insights from this figure, apart from the fact that more participants showed more adaptive behavior in the later runs, as indicated by the red and olive green bars in the figure. Participants’ strategies did not develop linearly, and it also did not converge to one specific type of behavior consistently within our experiment. To be able to see whether human learning had an influence on robot learning, we chose to remove the dimension of time (runs) from our participant data, and focus on whether and how a participant adapted over the whole experiment. We created the following clusters based on participant adaptation over the whole experiment:

• Does not adapt: participant shows no signs of adapting to strategies employed by the robot; participant either focuses on their own task, or constantly switches between behavior strategies as they focus too much on the robot.
• Adapts by balancing waiting and acting: participant shows signs that they adapt by waiting for the robot to act, and to use that robot behavior to determine their own response. It suggests that the participant understands that being passive for a while may cause the robot to act.
• Adapts by actively using *O2—passive large rocks* or *O3—breaking*: participant visibly adapts as they actively guide the robot to pick up or break rocks by waiting on top of those rocks.

This clustering of participants according to their dominant strategy resulted in three clusters with a similar number of participants per cluster, as shown in Table 4.

**TABLE 4:** The clusters resulting from manually clustering participants based on whether they adapted to the robot across the whole experiment.

Cluster	Participants
Does not adapt	2, 6, 9, 15, 21, 22, 23, 24 (n = 8)
Adapts by balancing passively waiting and acting	12, 13, 14, 16, 27, 28 (n = 6)
Adapts by actively using O2 or O3	3, 8, 10, 17, 19, 20, 26 (n = 7)

#### Combining Participant Adaptation and Robot Learning

**FIGURE 10:**
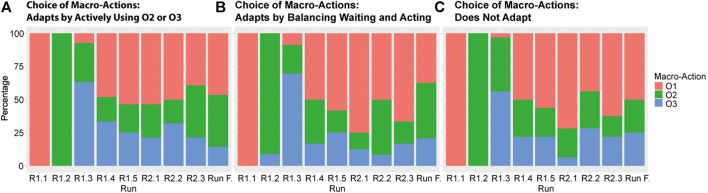
An overview of how often certain Macro-actions were chosen by the robot across all participants per run, split up by the level adaptation the participant showed: **(A)** shows participants who adapted by actively using *O2—passive large rocks* and/or *O3—breaking*, **(B)** shows participants who adapted by balancing waiting and acting, and **(C)** shows participants who did not adapt.

In order to explore whether these different types of adaptation employed by participants affected robot learning, and whether differences occur between clusters, we plotted the robot strategies per human adaptation cluster, as shown in Figure 10. These figures present the bar graphs of how often the different macro-actions were chosen across the group, in an attempt to more closely evaluate any possible differences between the three clusters. The figures suggest a response in the robots’ behavior to the participants’ actions, especially later on, in the final runs. When we compare the figures for “adapts by balancing passively waiting and acting” (Figure 10B) and “does not adapt” (Figure 10C), the former shows the trend of using *O1—picking up all* in the first level (first five runs) and using other strategies in the second level (run 6–8) more strongly. The figures for “adapts by actively using O2 or O3” (Figure 10A), however, shows that the robot already learned to use *O2—passive large rocks* and *O3—breaking* before being introduced with the second level, and actually moving back to *O1—picking up all* a little more toward the final runs.

This suggests that if participants learned more about the robot behavior and adapted their own behavior more strongly (by actively guiding the robot with *O2—passive large rocks* and *O3—breaking*), the robot was also able to learn to use those strategies more often. This combined learning effect therefore can be seen as learning at the team level.

## Discussion

### Interaction Patterns That Drive Co-Learning

We set out to investigate what interaction patterns between humans and robots drive co-adaptation as a precursor for co-learning. From the behaviors observed in our experiment, we identified a list of interactions and a set of interaction patterns. It should be noted, however, that (interactive) behavior is very much determined by the specific context. This means that our list should not be considered as a complete list of all possible co-adaptive interactions. It should rather be seen as a collection of interaction patterns that are likely to appear in contexts similar to ours, where co-adaptation is centered around mutual observation and harmonizing actions when collaborating.

The collection of interaction patterns can be used as a language for recording, analyzing and coding co-adaptive behavior. By describing observed behavior with such interaction patterns, complex behavioral observations of human-robot co-adaptive strategies can more easily be compared. The interaction patterns can also be useful as a vocabulary for a human-robot team itself to discuss the adaptations that they are engaged in, to help them elaborate and sustain successful collaborations over time.

### Validating the Research Environment

An important objective of this study is to improve our understanding of how human-robot co-learning develops, as well as how the adaptative processes of both partners interact. We defined claims for the experimental environment we designed; if these claims are justified, it means that it enabled us to study co-adaptation, the process that we consider to be a precursor for co-learning. Table 5 shows the claims described in *Claims: Expected Observations* and annotated conclusions as to whether we were able to justify the claims in the present study.

**TABLE 5:** The claims as presented in *Claims: Expected Observation*s that need to be justified in a co-adaptation experiment, including whether they were validated and an explanation of that conclusion.

Claim	Justified	Explanation
Different participants develop different ways of performing the task	Yes	When looking at the different interaction patterns that people engage in, and categorizations of their adaptive behavior, we can see that different people indeed performed the task in a variety of ways
The agent learns different sequences of strategy options for different participants	Partly	The results showed that not all agents learned the same model on an individual level. However, the models had much in common, suggesting that all agents learned similar behavior. When splitting this up in groups based on human adaptive behavior, there seems to be a difference in learned agent behavior between the different groups. Currently, however, we did not do any statistical analysis to test whether this is a significant result
Different teams converge to different ways of performing the task	Partly	When looking at the different interaction patterns that participants engaged in with their robot team partner, different teams solved the task in a variety of ways (see H1). However, it is unclear to what extent the robot contributed to this. Moreover, while participants generally gained more confidence in their strategy and expressed to experience a greater subjective collaboration fluency toward the end of the experiment, it is unclear to what extent the strategy of the team really converged to a stable one
The agent converges to a specific sequence of strategy options for most participants	No	While we did observe a logical development of the Q-values on a population level, this does not count for all of the individual agents. Moreover, it is not clear to what extent the agents really converged to a stable set of actions
The human converges to a specific strategy within the experiment	Partly	The categorizations of participant behavior show that participants settle on a stable strategy more and more over the course of the experiment. This is also shown by the development of the confidence scores and subjective collaboration fluency. True convergence to a stable strategy, however, is not clearly visible within the 8 runs of the experiment

As can be seen in the table, only one of the claims was justified completely. Fortunately, many of the other claims were partly justified. For the claims that we did not realize, the results provide cues for how to design a research environment that better fits the claims. We regard these findings as an important step toward studying and revealing the processes involved in human-robot co-learning. Aspects that should be improved upon or need further work center around a few problems that we will elaborate on below:

1. Behavior strategy: Convergence vs. flexible adaptations
2. Statistical analysis of complex behavioral data
3. Behavior of the individual team member vs. behavior of the team
4. Task effects vs. participant effects

In our claims in Table 7, we mentioned convergence several times. This stems from the principle that a Reinforcement Learning algorithm should aim for convergence toward an optimal solution. However, when studying co-learning, we specifically use dynamic task environments that have no fixed optimal solution, and in which unpredicted events can require strategy changes. In such environments, convergence is not a good criterion for performance, as agents (human as well as robot) are required to continuously learn and adapt. For human-robot co-learning it can be argued that it is better to make the algorithm learn certain repeated subsequences of interactions (or *interaction patterns*), and to store those in a rule-based manner. Once a pattern of interaction has proven to be successful in multiple instances of task situations, it can be applied, combined and if necessary revised in similar but other task situations. We therefore believe that future research into co-learning should not take convergence as a criterion for the robot’s behavior, but to focus on the emergence and sustainability of successful interaction patterns (aspect 1).

The results show that the robot had a similar learning process across all participants despite the high variety between individual participants. However, the behavioral data of both the robot and the participant is quite complex. Sometimes there are radical changes in behavior between one run and the next, and even within one run participants sometimes quite radically changed their behavior. It is a challenge to analyze such data as it is often difficult to clarify the origin of the behavior from the data. Our qualitative analysis and clustering is able to deal with this complexity and provides many useful insights, therefore we would advise future research into co-learning to include similar qualitative analyses. When further investigating co-learning, it will, however, also be relevant and interesting to verify insights statistically. This will require different design considerations. The current complexity in behavioral data is partly due to the interaction between two adaptive systems, and probably an inherent property of co-learning. Moreover, the human and robot can approach the task in many different ways by design. This property is a strength of our experiment, as it allows participants to behave relatively freely and naturally, but it also contributes to the complexity of the resulting co-adaptation. For future research, it will be important to explicitly take these properties into account when designing an environment for experimentation (aspect 2), in such a way that insights can be verified statistically. For example, in our design, the strategies of the robot were separate, nominal actions, but if we can design a learning agent such that their learned behavior is ordinal (e.g. by using more or less of a certain behavior), it might be easier to apply statistical methods. Moreover, we can look into data analysis methods used in complexity science, to see if they can be applicable to co-learning scenarios.

Lastly, it is currently a challenge to determine which aspects of the final team behavior are caused by adaptations of individual team members, and which by interactions between them. Similarly, we cannot yet conclusively determine which aspects of the learned strategy are caused by the task, and which by the individuality of a participant (e.g. what does the robot learn just because of the task, and what does the robot learn because a certain participant behaved a certain way). To solve this problem, we need to find ways to separate the different effects, for example by creating relevant baseline results. Letting the robot perform a task and learn by itself is not an option in the context of team tasks, as the nature of such task dictates dependencies between the team members. A possibility might be to create a simulated, possibly rule-based human agent for the robot agent to collaborate with.

### Future Steps for Studying Human-Robot Co-Learning

The discussion regarding the interactions that underlie co-learning provide several pointers and suggestions for improving the design of our research methods. Besides the suggestions above, however, there are several other directions in which we believe that co-learning research should develop. In this paper we developed an approach for studying co-learning. Since we were still defining what it means to study co-learning, the scope of the task, learning algorithm and opportunities for interaction had to be limited. Eventually, if we want to enable and study co-learning in full-fledged teams, it will be necessary to use more complex task environments, more intelligent agents or robots and more elaborate interaction and communication between the human and the robot. In the following section we will therefore further outline the two research directions mentioned in *Research Challenges* that we did not further address in this paper (research direction two and three).

The first direction is aimed at enabling the communication between partners within the team, especially communication about adaptations. We believe that in order for team members to produce successful sequences of interactive behavior, that can be used strategically across contexts, it is necessary that the team members can communicate with one another. The interaction patterns that we have identified might be used as a start for a vocabulary for such communication interactions, but the specific timing, modality and details of the interactions will have to be designed and studied.

The second direction is aimed at making the agent or robot more intelligent in terms of its abilities for co-learning. In the experiment we presented, our agent only learned based on task-related rewards. It makes sense to also explicitly reason about or take into account the human’s behavior and preferences in learning. There is a large body of research on personalizing robot behavior, e.g. by making the robot develop a user model of its partner, but these models often do not explicitly take into account that the human continuously learns and adapts. We therefore believe that there is a need for user models and team models that specifically accommodate the adaptive interactions as described in this paper. A team mental model that is able to represent the interactions within a team will support the partners in developing and sustaining successful adaptations and to synchronize and align their actions and learning processes.

## Conclusion

Co-learning is an important mechanism for building successful human-robot teams. However, there is no general understanding of what co-learning means in the context of human-robot teams. In this paper, we defined the concept of co-learning based on related literature, and positioned it in relation to co-adaptation and co-evolution. From this definition, it is clear that adaptive interactions between humans and robots play a central role in co-learning. We defined requirements for studying how bi-lateral adaptation emerges from the interactions between humans and robots.

From these requirements, we developed an experimental task environment based on a real-life Urban Search and Rescue task. The task was designed such that it allowed human participants to behave relatively naturally and freely, enabling us to record and analyze emerging adaptive interactions between a human and a robot. A bottom-up coding process based on Grounded Theory allowed us to identify recurring interaction patterns. The resulting list of interaction patterns describe stable situations (repeating subsequences of stable behavior) as well as sudden adaptations (changes in behavior happening over short periods of time). These patterns can emerge in similar task contexts, thereby forming a description and analysis method of co-adaptive behavior in human-robot teams.

Over the course of the experiment, the robot learned similar strategies for most participants. However, the results show that the learned strategies were slightly different depending on whether a human participant adapted their behavior to the robot. This suggests that human learning affected the robot’s Reinforcement Learning. More specifically, the human learning about and adapting to a specific strategy of the robot enabled the robot to learn to stick to that strategy. This shows how learning on the individual level lead to team level learning in our experiment.

This paper presents a theoretical framework and a methodological approach for studying the processes that underlie co-learning in human-robot teams. The strengths as well as the shortcomings of our approach provide ample directions for future research into this important process that ultimately defines the quality of human-robot teaming.

## Data Availability

The datasets presented in this study can be found in the following online repository: van Zoelen, Emma (2021), “Mutual Adaptation for Human-Robot Co-Learning - USAR task”, Mendeley Data, V1, doi:10.17632/r2y8z6bzg8.1.
