# Supplementary material for: Becoming Team Members: Identifying Interaction Patterns of Mutual Adaptation for Human-Robot Co-Learning
Source: Front Robot AI. 2021 Jul 6;8:692811. doi: 10.3389/frobt.2021.692811 (PMC8290358; doi:10.3389/frobt.2021.692811)
Supplement: Supplementary file 2 [file Table1.DOCX]

# Appendix A: Questions asked during the experiment

## Collaboration Fluency Questionnaire

- The human-robot team worked fluently together.
- The human-robot team’s fluency improved over time.
- The robot contributed to the fluency of the interaction.

These questions were evaluated on a slider ranging from 0 for ‘strongly disagree’ to 100 for ‘strongly agree’. The questions were taken from Hoffman, G. 2019. “Evaluating Fluency in Human–Robot Collaboration.” *IEEE Transactions on Human-Machine Systems* 49 (3): 209–18. https://doi.org/10.1109/THMS.2019.2904558.

## Interview Questions

Confidence interval:

- On a scale from 1 to 10, how confident are you that your strategy is the right strategy?

Interview questions:

- What was the strategy of the team in the end?
- Can you explain who did what in the task?
- Did the strategy change over the course of the experiment? How?
